# Commentary: Lung enteric-type adenocarcinoma with gastric metastasis: a rare case report and literature review

**DOI:** 10.3389/fimmu.2025.1598522

**Published:** 2025-09-25

**Authors:** Ganfa Huang, Chang Zhao

**Affiliations:** Department of Interventional Radiology, Guangxi Medical University Cancer Hospital, Nanning, Guangxi, China

**Keywords:** lung enteric-type adenocarcinoma, gastric metastasis, NRAS gene exon 3 mutation, chemotherapy and immunotherapy, non-small cell lung cancer

We read with great interest the case report by Li et al. entitled “Lung enteric-type adenocarcinoma with gastric metastasis: a rare case report and literature review” (1). The authors present the first documented case of pulmonary enteric-type adenocarcinoma (ETAC) demonstrating gastric antral metastasis, providing valuable insights into the biological behavior of this rare malignancy. While the comprehensive documentation of diagnostic procedures (including imaging, histopathological analysis, and molecular profiling) and therapeutic strategies establishes an important clinical reference, we would like to address several methodological considerations regarding the imaging surveillance protocol.

**Figure 1:**
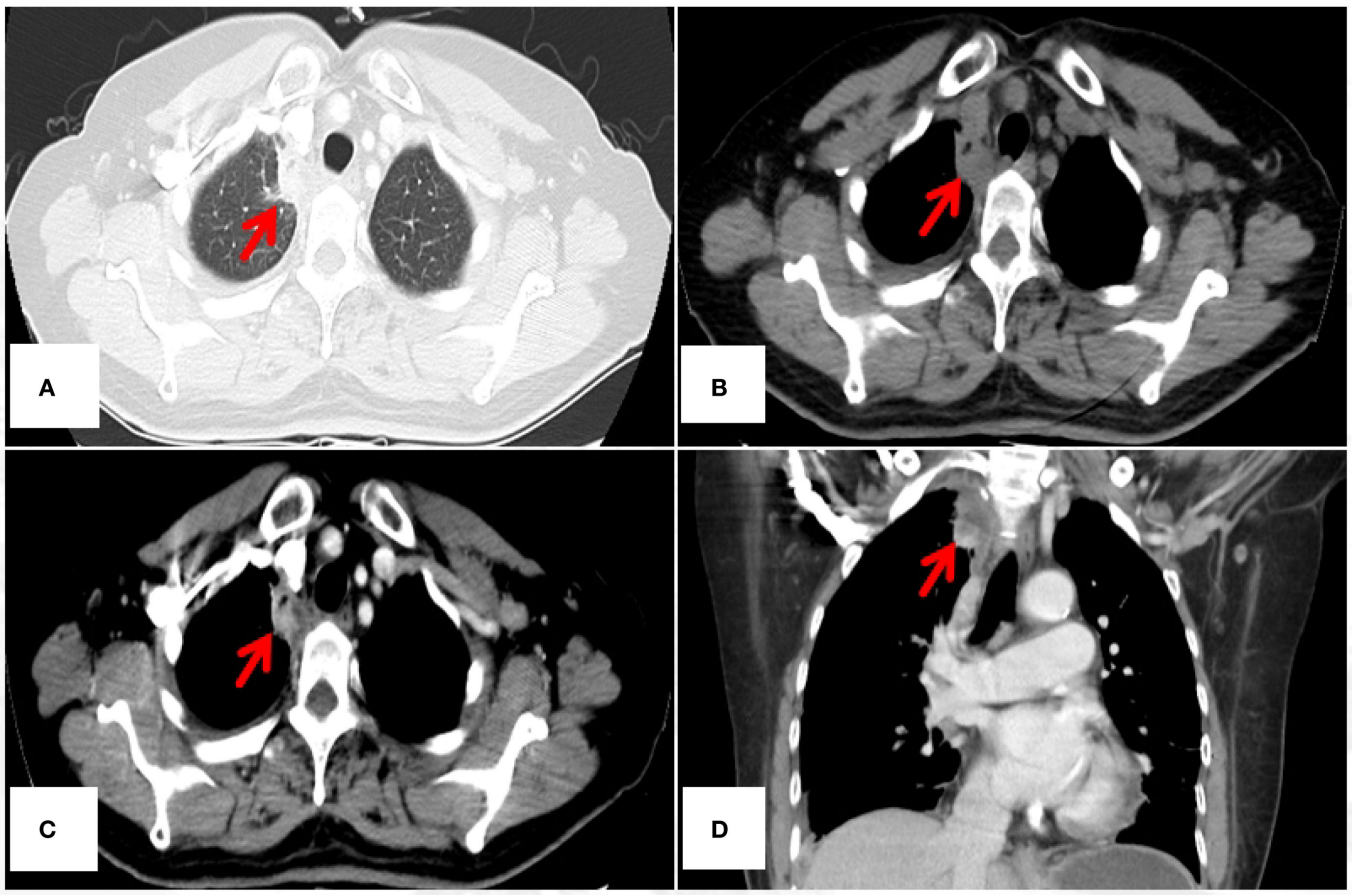
45-year-old female diagnosed with ETAC. Immunocytochemistry shows: Ber - EP4(+), CK7(+), TTF - 1(SPT24)(-), Napsin A(-), Villin(+), CDX - 2(+), GATA3(-), PAX8(-), CR(-). **(A)** Lesion under lung window (arrow); **(B)** Lesion under mediastinal window non – contrast (arrow), **(C, D)** Lesion under mediastinal window contrast - enhanced, showing heterogeneous enhancement(arrow). CT, computed tomography.

The authors used only non-contrast-enhanced CT (NCECT) for postoperative surveillance from July 2021 to March 2024. Of particular concern is that, for malignant lesions demonstrating “heterogeneous enhancement” on contrast-enhanced CT (CECT) as documented in this ETAC case, the inherent limitations of NCECT in soft tissue characterization may substantially reduce diagnostic specificity. Take a case of ETAC in our center as an example (see **Figures 1A-D**). The vascular heterogeneity and necrotic components characteristic of malignant tumors produce distinctive enhancement patterns on CECT, whereas both postoperative changes (e.g., fibrotic remodeling, atelectasis) and inflammatory processes manifest as isodense shadows on NCECT. This makes it challenging to differentiate tumor recurrence from benign structural alterations. Current evidence from multiple studies demonstrates the superior diagnostic performance of CECT in detecting locoregional recurrences (including hilar, mediastinal, and pleural recurrences) during postoperative surveillance of pulmonary malignancies (2–4). In the present case, exclusive reliance on NCECT for assessing disease progression (August 2023) and monitoring response to PD-1 inhibitor therapy (October 2023 and March 2024) may preclude accurate characterization of soft tissue density biology (viable tumor versus scar tissue versus inflammatory changes), potentially confounding the objective interpretation of therapeutic outcomes.

Thus, the following recommendations are put forward: firstly, if CECT is contraindicated (e.g., due to renal insufficiency or uncontrolled hyperthyroidism) or omitted per the institutional protocol, this should be clearly stated in the discussion section to enhance methodological transparency. This is because we found that at the time of the first CT examination in the articles, the patients were eligible for CECT, which also yielded good imaging. Secondly, for lesions with distinct CECT enhancement features, the monitoring protocol should prioritize CECT within the first two critical postoperative years to help differentiate recurrence from confounding factors like elevated diaphragm or pleural thickening. This aligns with the method used by Guo et al. (5), who dynamically assessed lesion morphology and enhancement patterns during treatment monitoring using consecutive enhanced CT scans. Thirdly, subsequent follow-up examinations using NCECT should include clear technical limitations in the graphic legends and discuss their potential impact on clinical interpretation.

This landmark study provides the first documentation of ETAC’s gastric metastatic potential, carrying significant implications for clinical practice. Our suggestions aim to emphasize the critical importance of precision imaging in monitoring rare malignancies while encouraging methodological optimization in future investigations.
